# Cognition-tracking-based strategies for diagnosis and treatment of minimal hepatic encephalopathy

**DOI:** 10.1007/s11011-020-00539-w

**Published:** 2020-06-03

**Authors:** Weijia Han, Huanqian Zhang, Ying Han, Zhongping Duan

**Affiliations:** 1 Difficult & Complicated Liver Diseases and Artificial Liver Center, Beijing Youan Hospital, Capital Medical University, Beijing, 100069 China; 2 Beijing Municipal Key Laboratory of Liver Failure and Artificial Liver Treatment Research, Beijing, China; 3 Yidu Central Hospital of Weifang Medical College, Shandong, China; 4 Department of Immunologic Liver Disease, Beijing YouAn Hospital, Capital Medical University, Beijing, China

**Keywords:** Cognitive impairment, Diagnosis and treatment, Minimal hepatic encephalopathy, Nerve cells

## Abstract

Minimal hepatic encephalopathy (MHE), which shows mild cognitive impairment, is a subtle complication of cirrhosis that has been shown to affect daily functioning and quality of life. However, until 2014, relevant guidelines do not give much attention to the diagnosis and treatment of MHE, resulting in patients being ignored and denied the benefits of treatment. In this review, we summarize recent cognition-based research about (1) alteration of nerve cells, including astrocytes, microglial cells and neurons, in mild cognitive impairment in MHE; (2) comparison of methods in detecting cognitive impairment in MHE; and (3) comparison of methods for therapy of cognitive impairment in MHE. We hope to provide information about diagnosis and treatment of cognitive impairment in patients with MHE.

## Introduction

Minimal hepatic encephalopathy (MHE), which affects 30–55% of cirrhosis patients, is a subtle complication of cirrhosis that may have a detrimental effect on daily functioning. In the MHE phase, patients show mild cognitive impairment, which lead to deficits of attention, and psychomotor slowing (Arias et al. 2015; Metwally et al. 2019).

However, only half of clinicians had studied their cirrhosis patients for MHE, and 38% had never screened patients for MHE (Ridola et al. 2018). Until 2014, relevant guidelines did not recommend routine screening for MHE nor were the patients treated except on a case-by-case basis. Patients with MHE might be ignored and denied the benefits of treatment. However, researchers have shown the need to screen and treat patients with neurocognitive impairment associated with MHE (Vilstrup et al. 2014).

MHE is often undiagnosed for the following reasons: (1) diagnostic criteria have not been standardized; (2) there is no overt clinical manifestation in patients with MHE; and (3) hyperammonemia is present with inflammation and certain levels of ammonemia (Felipo et al. 2012; Jiao et al. 2017; Stinton and Jayakumar 2013). In terms of treatment, not all clinicians have already applied the treatment of MHE patients, because the treatment of MHE has not been included in the guidelines (Vilstrup et al. 2014).

Depending on technological progress, researchers have introduced cognition-based methods to detect and treat MHE. In this review, we summarize: (1) the relationship between cognitive impairment and nerve cell injury; (2) comparison of diagnostic methods for MHE; and (3) comparison of therapeutic methods for MHE.

## Nerve cell injury in MHE: cognition impairment

During MHE, the main contributor to cognitive impairment is hyperammonemia. In patients with liver disease, ammonia accumulates in blood due to deficient activity of hepatic urea cycle enzymes. In the brain, nerve cells take up ammonia and cause cognitive impairment. For example, astrocytes take up ammonia and glutamine synthetase detoxifies ammonia into glutamine. The rapid accumulation of glutamine creates an osmotic gradient that results in astrocyte swelling (Stravitz et al. 2018).

**Fig. 1:**
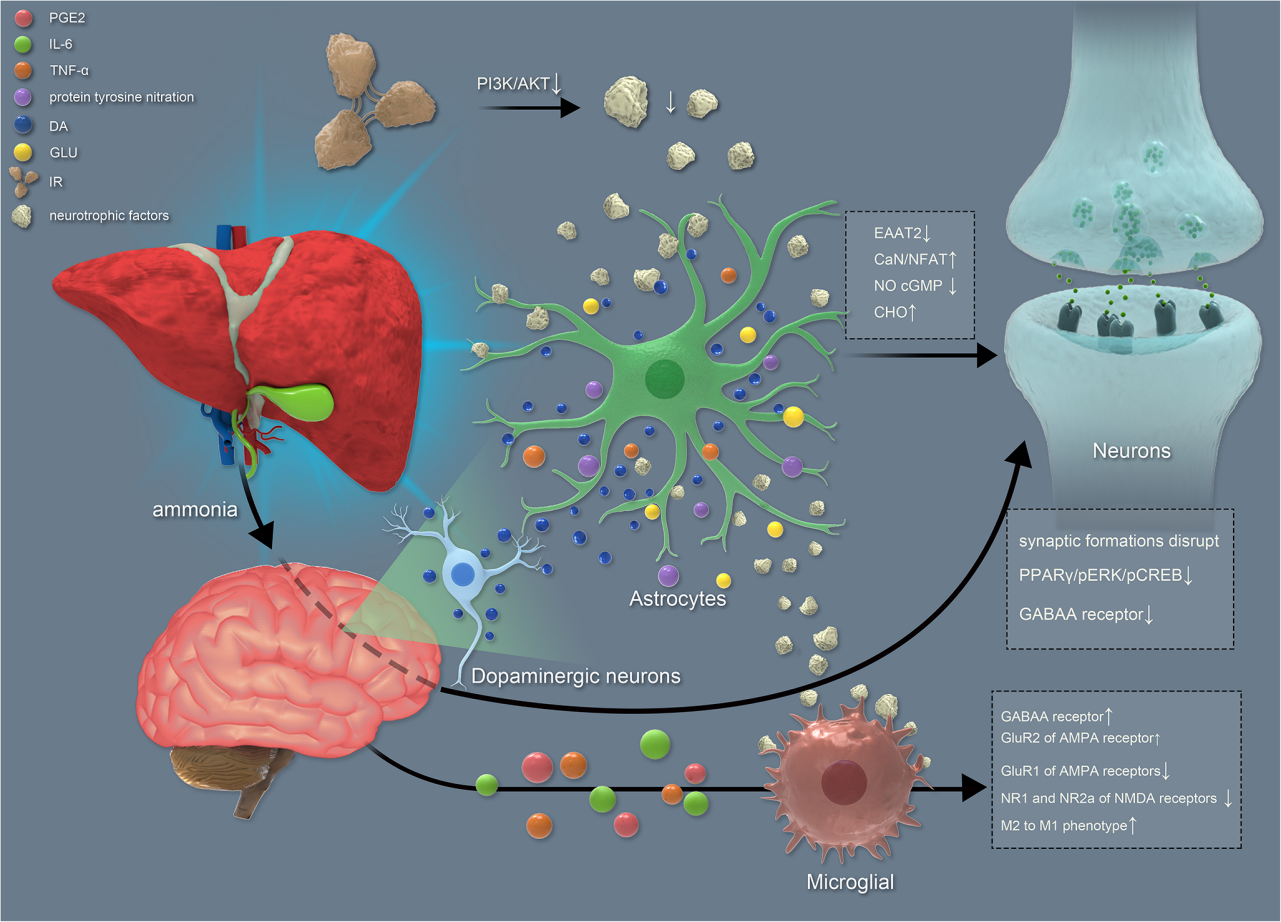
MHE caused by hyperammonemia. Ammonia through the blood brain barrier into the brain, promote DA release. Then, DA promotes increased extracellular GLU and astrocytes change, such as EAAT↑. Furthermore, it can lead to neural apoptosis. DA can also lead to increased release of inflammatory mediators, such as IL6, which can lead to signal transduction alteration, such as GABBA receptor↑. These alterations can lead to neuron damage. Ammonia can also directly cause signal transduction changes and neuron damage.IR also can lead to neurotrophic factors production ↓, which impaired astrocytes. DA: dopamine; GLU: glutamine; IL: interleukin

Hyperammonemia and the changes caused by its downstream damage, such as changes in dopamine (DA) secretion, play important roles in nerve cell impairment. For example, hyperammonemia significantly reduces long-term potentiation and alters mRNA for DA receptors, which cause deficits in disturbed synaptic plasticity and novelty acquisition in hippocampal and corticostriatal pathways involved in goal-directed and learning behavior (Chepkova et al. 2017). Besides, the alteration of Gama-aminobutyric acid (GABA) also plays important roles in nerve cell impairment. GABA is the inhibitory nerve conduction in the brain. When the brain is over-excited, GABA can play the restrain role and eliminates the state of anxiety. Anxiety, insomnia, fatigue, anxiety and other symptoms can occur when GABA is lacking. Hyperammonemia is associated with increased membrane expression of the GABA transporter. Increased GABAergic tone in the cerebellum impairs motor coordination and learning ability in the Y maze. In this part, we summarize the changes in nerve cells caused by hyperammonemia (Fig. 1).

### Astrocytes atrophy

Astrocytes, which actively involved in dynamic signaling in the central nervous system, are the predominant type of glial cells. Astrocytes participate in a variety of essential physiological processes, including glucose metabolism, glutamate clearance, and ionic homeostasis. Astrocytes also contribute to memory formation and information processing in the brain (Dossi et al. 2018; Jackson and Robinson 2018).

Under the influence of DA, astrocytes undergo pathological changes and affect the process of MHE. In MHE, DA stimulates production and secretion of astrocytic tumor necrosis factor (TNF)-α. Astrocytic TNF-α, which triggers neurodegenerative progression consequently or indirectly, resulting in cognitive impairment. Ding et al. found that low-dose DA (10 μM) produced TNF-α in primary astrocytes, and co-culture of these astrocytes and neurons exhibited neuronal apoptosis compared with control group (Ding et al. 2016). Ding et al. also demonstrated that DA induced astrocytic protein tyrosine nitration, which was attributed to NADPH oxidase subunits and induced reactive oxygen species production and p47(phox) phosphorylation, but decreased neuronal-type NO synthase expression (Ding et al. 2014).

DA can cause alteration of pathways localized in astrocytes, which can lead to cognitive impairment in MHE. For example, DA activates trace amine-associated receptor 1 to down-regulate excitatory amino acid transporter-2 in astrocytes and increase extracellular Glu levels, which interact with α-amino-3-hydroxy-5-methyl-4-isoxazolepropionic acid receptor. DA and Glu cause memory impairment through activation of calcineurin/nuclear factor of activated T cells signaling in MHE (Ding et al. 2017a).

Insulin resistance (IR), which often occurs in cirrhosis patients, contributes to the MHE pathogenesis. Ding et al. found that the final production of neurotrophic factors, including PI3K/AKT signaling pathway to the phosphorylation of N-Methyl-d-Aspartate receptors and downstream activation of the CaMKIV/CREB pathway, was impaired in MHE rats (Ding et al. 2018).

### Microglial alteration

Microglia are critical for developmental processes and maintenance of neural homeostasis. In response to injury or insult, microglia acquires complex and diverse phenotypes, allowing them to participate in cytotoxic responses as well as in immunoregulation or injury resolution. Detrimental microglial phenotypes are produced by pro-inflammatory cytokines interleukin (IL)-1β, Toll-like-receptor (TLR)-4 agonist lipopolysaccharide, and TNF-α. These pro-inflammatory microglia are characterized by immune-potentiating abilities, antigen-presenting and microbicidal (Fumagalli et al. 2018).

Hyperammonemia caused reversible and rapid induction of peripheral inflammation, with increased TNF-α, pro-inflammatory prostaglandin (PG)-E2 and IL-6. And hyperammonemia also caused reduced Anti-inflammatory IL-10 and microglia activation in hippocampus. Increased TNF-α and IL-1β levels and phosphorylation (activity) of p38 cause GABAergic and glutamatergic neurotransmission. For example, membrane expression of GluR2 of AMPA receptor and subunits α1 of etc. GABAA receptor is increased, while expression of subunits GluR1 of AMPA receptors and NR1 and NR2a of NMDA receptors is reduced. These altered membrane expression receptors are associated with hyperammonemia-induced microglial activation. And are responsible for impairment of spatial learning and altered neurotransmission in the radial maze. In turn, these inflammatory injuries contribute to microglial differentiation from anti-inflammatory M2 to pro-inflammatory M1 phenotype (Agusti et al. 2011; Balzano et al. 2019; Hernandez-Rabaza et al. 2015; Hernandez-Rabaza et al. 2016).

### Neurons injury

In the brain, most neurons are generated during embryogenesis and are not replaced frequently. These neurons need to live for the lifetime of the individual, because they were unable to remove dysfunctional proteins and organelles. So, it is exceptionally vulnerable for neuronal proteins and organelles to overuse and damage (Kulkarni and Maday 2018).

Cholinesterase (CHO) is involved in the perturbation of neural synaptogenesis. Cholinesterase (CHO) overload in combination with DA burden elicits memory loss and cognitive decline via the peroxisome proliferator-activated receptor (PPAR) γ/extracellular signal-regulated kinase (ERK)/CREB pathway in MHE. In turn, DA triggers CHO biosynthesis via activation of the C-Jun N-terminal kinase 3/sterol regulatory element-binding protein 2 signaling pathway in primary cultured astrocytes. Zhuge et al. found that CHO secreted from astrocytes stimulated secretion of DA from primary cultured neurons. PPARγ/pERK/pCREB expression was decreased by DA-induced synergistic leads to synergistic synaptic impairment in (Zhuge et al. 2019).

### Neuroinflammation

Neuroinflammation in hippocampus impairs memory and spatial learning. Neuroinflammation in cerebellum impairs learning in a Y maze and motor co-ordination. Hyperaminemia triggers neuroinflammation by activating microglia and increasing markers associated with impaired cognitive function (Cabrera-Pastor et al. 2019; Malaguarnera et al. 2019). Cabrera-Pastor demonstrated that hyperammonemia induced neuroinflammation was related to impaired memory and spatial learning in hyperammonemic rats. Chronic hyperammonemia also increased the level of IL-1β. Increased IL-1β level alters neurotransmission and impairs spatial learning (Cabrera-Pastor et al. 2016; Hernandez-Rabaza et al. 2015). Balzano et al. demonstrated that hyperaminemia caused the elevation of pro-inflammatory factors and IL. This alteration was associated with glutamate receptors membrane expression and impairment of spatial memory (Balzano et al. 2019).

## Diagnosis of MHE: cognitive evaluation

### Psychometric tests

Many new psychometric tests have emerged in recent years, such as psychometric hepatic encephalopathy score (PHES) (Ferenci et al. 2002), Stroop app (Bajaj et al. 2013), and animal naming test (ANT) (Campagna et al. 2017). Ideal screening tests for MHE should be characterized by less time-consuming, objective outcome, independent of specialists’ interpretation, simple to use and free from copyright and fees (Yoon et al. 2019).

#### PHES

PHES comprises of five tests and has been recognized as the gold standard for diagnosis of MHE since 1998. It is widely accepted that diagnostic of MHE is PHES score ≤ −5 (Ferenci et al. 2002; Luo et al. 2019; Singh et al. 2016). Tsai et al. found that PHES can be a useful tool for detecting patients with MHE in around one third of outpatients with cirrhosis (Tsai et al. 2015). Badea et al. showed the prevalence of MHE in Romanian cirrhosis patients. PHES of the healthy control group was significantly higher than that in the liver cirrhosis group (Badea et al. 2016). But Coskun et al. set −4 as the cutoff in Turkish PHES nomograms. They found that the PHES score in the cirrhotic group was −2.18 ± 3.3, which was significantly lower than the center of gravity (Coskun et al. 2017). To validate widely applicable norms, future multicenter studies are needed.

#### Stroop app

The Stroop app score, which is based on ipad or ipod, is significant for diagnosis of MHE (Bajaj et al. 2013). The method is shown in Table 1. The Stroop app has a accuracy rate of 0.74 and is suitable for MHE screening (Yoon et al. 2019). Zeng et al. demonstrated that >97.34 s for “Off” state time and > 186.63 s for “Off” state + “On” state time had the maximum area under the curve values in all patients. Meanwhile, “Off” state + “On” state time had the highest sensitivity with a cutoff of 186.63 s. Compared with PHES, the Stroop app is time saving, accessible, convenient, and accepted by patients and clinicians (Zeng et al. 2019).

**Table 1:** Comparison of different kinds of diagnostic methods

		Method	Disadvantage
Psychometric tests	PHES	It comprises the number connect test (NCT)-A, NCT-B, serial dotting test, line tracing test, and digit symbol test, which are each scored from 1 to −3	☆take time; ☆affected by demographic factors; ☆lack ecological validity language functions; ☆only two cognitive domains
	Stroop app	“Off” and “On” task are the two components depending on the discordance or concordance of the stimuli. Patients have 2 training runs for two components. In “Off” state, pound signs (###) presented in red, green or blue, one has to respond as quickly as possible by touching the matching color, which were also randomized and not fixed to their respective positions. This continues until a total of 10 presentations. If the subject makes a mistake, the run stops and has to restart again. The “Off” state continued till the subject had achieved 5 correct runs. In the “On” state, the patient has to touch the color of the word presented which is actually the name of the color in discordant coloring. The test of cognitive processing controlling for psychomotor speed was subtracting the “Off” state time from the “On” state time	☆complex ☆optimal cutoff is varied
	ANT	In ANT test, patients were asked to list as many animals as they could in 1 min. All repetitions and errors were excluded from the calculations	☆no significant limitatin
	Other	ICT: An alcohol-related or control picture was presented in the centre of the screen with one of two letters superimposed on one of the four corners of the picture. Patients were instructed to press the space bar if the go cue was present, but to withhold their response if the no-go cue was present. During each trial, the picture and letter remained on screen until the participant responded or until a 1500 ms timeout had elapsed eNCT: electronic based NCT	☆ICT requires highly functional patients
CFF		Patient is equipped with a light shade, and the red light spot flashes at a ratio of 1:1 at a frequency of 60 Hz. At the beginning, the subject cannot recognize the flicker because the flicker frequency is fast, and then the flicker frequency gradually slows down until the subject can recognize the flicker stop.	☆expensive; ☆time-consuming; ☆dependent on the specialist’s interpretation
MRI		Marker, including mean kurtosis values, Six ROIs, ALFF values and default mode network, are useful biomarkers for MHE detection.	☆lack of detection accuracy of the measured signal
inflammatory cytokines		Na	☆no studies on the accuracy and sensitivity of its application

#### ANT

Cognitive functions related to prefrontal anterior/ cortex cortical areas are sensitive to the ANT. Patients are asked to list as many animals as they can in 1 min. Errors and repetitions should be excluded from the calculations (Table 1). Campagna et al. found that, in order to eliminate age interference, a simplified ANT was obtained, adding 3 animals for patients with <8 years of education and 6 animals if they were over 80 years old in addition (Campagna et al. 2017). Labenz et al. demonstrated that the simplified ANT may become an initial screening tool for assessment of MHE. They found that ANT was Significant lower in patients with MHE. The best discrimination between patients with and without MHE is naming <20 animals. But when the cutoff value is ≥23 animal names, 38.5% of patients could be avoided further testing for MHE, and the negative predictive value was 84% (Labenz et al. 2019).

#### Other tests

The inhibitory control test (ICT) represents the ability to suppress irrelevant motor or cognitive processes, and is useful for diagnosis of MHE (Di Lemma and Field 2017; Hartmann et al. 2019). The method is shown in Table 1. Gupta et al. demonstrated that ICT is correlated with disease severity, predicts the development of HE, and has excellent test–retest reliability. ICT was considered abnormal when there were ≥ 14 ICT lures. Mean ICT lures were higher and target accuracy was lower in cirrhotic patients with MHE than those without MHE. ICT had a sensitivity of 92.6% and specificity of 78.5% with an area under the receiver operating characteristic curve of 0.855 for MHE (Gupta et al. 2015).

The novel electronic number connection test (eNCT) has test–retest reliability to detect cognitive function and monitor cognitive impairment in patients with cirrhosis. Wuensch et al. found that the eNCT performance was negatively correlated with PHES performance in patients with cirrhosis. Control participants showed significantly faster eNCT completion times compared with cirrhosis patients (Wuensch et al. 2017).

### Critical flicker frequency (CFF) and electroencephalography (EEG)

CFF reflects dysfunction of nerve conduction in the brain. It is an objective test that avoids the deviation caused by cultural differences. The CFF method is shown in Table 1 (Wang et al. 2013). CFF is widely acknowledged as an adjunct tool for diagnosis of MHE. However, some researchers believe that CFF should be used as an adjunct to the PHES test because of its low sensitivity for detecting MHE. They found that CFF had a diagnostic accuracy of 70.6%, specificity of 82%, sensitivity of 39% for detecting MHE (Ozel Coskun and Ozen 2017). However, for the threshold of CFF, the researchers had different choices. Barone et al. evaluated that a CFF cut-off of 39 Hz is effective in predicting survival and the first episode of MHE in cirrhosis patients who had never experienced MHE. With progression of the Child–Pugh class, the prevalence of CFF ≤39 Hz significantly increased (Barone et al. 2018). Greinert et al. found that most patients with a MELD score > 24 and CFF <43 Hz had MHE. They demonstrated that combination of CFF and MELD score may be used as a first diagnostic step to filter patients, in whom further MHE testing could be avoided. Specificity and sensitivity of a CFF cut-off of 43 Hz was 93.5% and 42.9%. (Greinert et al. 2016).

EEG has been acknowledged to confirm the presence and predict the severity of HE. Recently, Olesen et al. found increased sample entropy of the EEG in patients with MHE. α activity is gradually replaced by slowed brain oscillations typically with θ in the frequency range (4–8 Hz) in the transition from an unimpaired mental state to HE (Olesen et al. 2016). Spectral EEG is a quantitative tool for diagnosis and assessment of the response to treatment in MHE. Spectral EEG analysis showed lower mean dominant frequency and higher θ relative power but lower α relative power in patients with MHE than in patients without MHE. Singh et al. found that with spectral EEG, 96% sensitivity, 84% specificity and 90% accuracy were obtained for diagnosis of MHE (Singh et al. 2016).

### Imaging

Magnetic resonance imaging (MRI) applies electromagnetic waves emitted by a graded magnetic field to acquire the internal structure of the objects (Lockwood et al. 1991; Shawcross et al. 2007). The biomarker for MHE is altered regional brain spontaneous activity. For example, the mean kurtosis values in the putamen decrease in the globus pallidus, putamen, caudate nucleus, and/or thalamus in patients with MHE (Sato et al. 2019). Chen et al. demonstrated that the amplitude of low frequency fluctuation (ALFF) values within six regions of interest (ROIs) correlated with PHES in cirrhosis patients. The ROIs contains the posterior cingulate cortex/precuneus, bilateral medial frontal cortex/anterior cingulate cortex, right lingual gyrus, the left precentral and postcentral gyrus, inferior/superior parietal and middle frontal gyrus lobule (Chen et al. 2016). Zhong et al. also found that patients with MHE had significant decreased ALFF (Zhong et al. 2016). Default mode network function, especially the medial prefrontal cortex, might also be a useful imaging marker for differentiating MHE from cirrhosis. The MHE patients showed even more decreased connectivity in medial prefrontal cortex, left superior frontal gyrus, and right middle temporal gyri when compared with non-MHE patients (Qi et al. 2014).

Different kinds of MRI play an important role in the detection of MHE. Kooka et al. demonstrated magnetic resonance spectroscopy, which shows that reduced magnetization transfer ratio in the whole brain field and an increase in glutamate/glutamine or taurine in chronic HE patients contribute to early and objective diagnosis of MHE. The levels of brain glutamine were significantly lower and the levels of brain myo-inositol were significantly higher in the control group compared with the MHE group (Kale et al. 2006; Kooka et al. 2016; Rovira et al. 2001). Altered diffusion kurtosis imaging metrics indicate brain microstructure abnormalities in MHE patients. Significantly alterations in axial diffusivity, radial diffusivity, and MD in a wide range of regions, including corpus callosum, left thalamus, were closely correlated with cognitive score (Li et al. 2019). Diffusion tensor imaging can differentiate MHE from non-MHE patients. Chen et al. found that in MD or fractional anisotropy maps, two spatially distributed white matter regions yielded 75.4–81.5% and 83.1–92.3% classification accuracy when differentiating patients with and without MHE (Chen et al. 2015).

### Inflammatory cytokines

In addition to hyperammonemia, inflammation also modulates neuropsychological function in patients with MHE. For example, Circulating IL-6 is negatively associated with memory function in low-dose endotoxemia (Krabbe et al. 2005; Tsai et al. 2015), serum IL-6 and IL-17a levels are independent risk factors for MHE in HBV-infected patients (Li et al. 2015), IL-1β is a potential, independent predictor of MHE (Wunsch et al. 2013), patients with MHE have significantly higher TNF-α is significant higher in MHE patients (Srivastava et al. 2011). These inflammatory cytokines may become biomarkers for MHE diagnosis, but further researches are needed.

### Comparison

Paper-and-pencil test used in PHES is the gold standard for diagnosis of MHE. But the process of diagnosis of PHES is inconvenient and affected by many factors. The diagnostic methods take time, and are affected by demographic factors, and lack ecological validity and language functions, such as verbal memory. PHES focuses on only two cognitive domains but it is not sensitive enough to detect early neurological alterations. Patients classified as without MHE by PHES have a high risk of suffering overt HE. Around 40% of patients without MHE according to PHES fail two other psychometric tests (Bajaj 2008 a; Gimenez-Garzo et al. 2017; Nardone et al. 2016; Seo et al. 2017; Wang et al. 2008). Other kinds of psychometric tests also have limitations. The ICT, which is a computerized test of response inhibition and working memory, requires highly functional patients. The Stroop app, which evaluates psychomotor speed and cognitive flexibility, is also a complex task that is applicable in highly functional patients (Bajaj et al. 2013). Tapper et al. conducted a meta-analysis to evaluate different kinds of psychometric tests. They compared ICT, Stroop app and ANT in diagnosis of MHE. They found that optimal cutoff for the Stroop app still varies. Good performance in the ICT, Stroop app or ANT is related to reduced development of HE, but longitudinal data are still limited. Studies are needed in clinically representative populations with cutoffs validated (Tapper et al. 2018). However, psychometric tests are irreplaceable now because MHE has subtle abnormalities that can be detected only using specific neuropsychometric and/or neurophysiological tools in cirrhosis patients with otherwise normal neurological examination results (Stewart and Smith 2007). Among all psychometric tests, the ANT is reasonably widespread in humans of every culture, and the influence of gender, age and education, if any, might be limited (Campagna et al. 2017).

CFF is a noninvasive, rapid, simple test for diagnosis of MHE. Compared with PHES, CFF has a positive predictive value of 93.2 ± 7.44%, specificity of 92.7 ± 7.96%, negative predictive value of 90.4 ± 8.91% and sensitivity of 91.1 ± 8.32%. CFF is excellent for diagnosis of MHE, with an area under the curve of 0.937 (Metwally et al. 2019). At one time, scientists thought EEG was an important method to predict MHE, but the detection of MHE showed limited agreement between PHES and EEG (Nardone et al. 2016). Meanwhile, it is difficult to make good use of EEG and CFF, as they are expensive, time-consuming, and dependent on specialist interpretation (Yoon et al. 2019). CFF is recommended as an adjunct (but not replacement) to psychometric testing.

Neuroimaging studies can detect diffuse abnormal metabolic activity of nerve cells, which is a typical feature of patients with MHE. MRI can provide objective and reliable imaging biomarkers that are necessary to help diagnose or identify MHE (Zhang et al. 2014). One major drawback of MRI concerns the lack of detection accuracy of the measured signal, but with technical advances, a solution to that problem is imminent (Janssen et al. 2018).

In addition to hyperammonemia, there is a parallel relationship between inflammatory cytokines and MHE, or a significant correlation between proinflammatory cytokines with MD values on diffusion tensor imaging (Srivastava et al. 2011) or PHES (Wunsch et al. 2013). However, there have been no studies on the accuracy and sensitivity of its application in the diagnosis of MHE, and more studies are expected.

Recent guidelines suggest that either alternative techniques, such as computerized tests, neurophysiological testing or EEG should be used alongside PHES for multicenter studies (Morgan et al. 2016; Vilstrup et al. 2014) (Table 1).

## Treatment of MHE: cognition recovery

### Prevent nerve cells

As mentioned above, nerve cell injury plays an important role in MHE progression, so protection of nerve cells is one way to prevent and treat MHE. Good nutritional status is an important way to relieve nerve cell damage. Myosteatosis and sarcopenia, probably by reducing the handling of ammonia in the muscle, are independently associated with MHE. Venous ammonia is significantly higher in patients with sarcopenia and myosteatosis and inversely correlated with both parameters (Nardelli et al. 2019). Wnt5a can reverse the decrease in spatial learning and memory in an MHE rat model. Down-regulation of neurotrophins, which are synthesized by Wnt5a, inhibits the interaction between Wnt5a and Frizzled-2 in astrocytes in MHE (Ding et al. 2017b).

Reducing neuroinflammation is also an important way to relieve nerve cell damage. Malaguarnera et al. demonstrated that bicuculline decreases anxiety and improves working memory and spatial learning in hyperammonemic rats. Bicuculline can reduce activation of GABAA receptors, which contributes to neuroinflammation. Meanwhile, bicuculline reduces astrocyte activation and not microglial activation. Bicuculline reverses the changes in membrane expression of AMPA and NMDA receptor subunits (Malaguarnera et al. 2019). Sulforaphane also can be useful in reducing neuroinflammation, normalizing membrane expression of glutamate and GABA receptors, restoring spatial learning, and improving cognitive function in cirrhosis patients with MHE, and promoting microglial differentiation from M1 to M2 phenotype and reducing activation of astrocytes in hyperammonemic rats (Hernandez-Rabaza et al. 2016). Sildenafil normalizes TNF-α and IL-1β levels, p38 phosphorylation, and membrane expression of NMDA, GABAA and AMPA receptors and restores spatial learning (Hernandez-Rabaza et al. 2015). Meanwhile, p38 inhibitor SB239063 can reduce inflammatory markers, as well as microglial activation. Agusti et al. showed that treatment with SB239063 completely restores coordination, motor activity, and learning ability in PCS rats (Agusti et al. 2011). Anti-TNF-α, which does not cross the blood–brain barrier, prevents hyperammonemia-induced neuroinflammation, alterations in microglial activation and cognitive impairment. This is also associated with altered membrane expression of glutamate receptors and impairment of spatial memory (Balzano et al. 2019).

### Regulate intestinal flora

Disorder of intestinal flora and bacterial translocation, which increase production and absorption of intestinal toxins, are closely related to HE. There are different degrees of intestinal flora disorder in patients with chronic liver disease. Beneficial bacteria such as bifidobacteria are decreased while urease-producing bacteria are the source of gut-derived toxins. Production and absorption of intestinal toxins significantly increase, but the liver cannot metabolize these toxins completely, which leads to toxin retention (McPhail et al. 2010; Sanchez et al. 2005).

MHE is also associated with individual microbiota signatures. For example, the relative abundance of Lactobacillaceae is higher in MHE, whereas abundance of autochthonous Lachnospiraceae is higher in those without MHE (Bajaj et al. 2019). Therefore, the adjustment of intestinal flora structure can also become an important way to treat MHE. For example, Xia et al. found that MHE patients’ cognition was significantly improved after probiotic treatment. In the probiotics-treated group, *Enterococcus* and Enterobacteriaceae were significantly decreased while the predominant bacteria were significantly enriched (Xia et al. 2018). Probiotic, rifaximin, L-ornithine L-aspartate (LOLA) and lactulose are important for improving intestinal flora, which is associated with reduction in ammonia.

#### Probiotics

Probiotics are well-tolerated, natural and safe and appropriate for long-term treatment of MHE (Jiang et al. 2015). Probiotics can improve the parameters of the intestinal mucosal barrier, which might have contributed to decreased ammonia levels and improved cognition. Bajaj et al. demonstrated a significant rate of MHE reversal and excellent adherence in cirrhosis patients after probiotic yogurt supplementation with potential for long-term adherence. In their randomized controlled trial (RCT), patients taking yoghurt had an improved PHES score compared to baseline/no treatment group (Bajaj et al. 2008 b). In a meta-analysis of RCTs of the effects of probiotics on serum ammonia, endotoxin, and MHE, Cao et al. showed that probiotics were more likely to reduce values in the NCT, improve MHE, and prevent HE progression (Cao et al. 2018). However, short-term administration of probiotics when compared with placebo did not produce any significant improvement in patients with MHE. Saji et al. found no significant change in the parameters such as arterial ammonia, evoked responses and NCT before and after treatment with short-term probiotics when compared to placebo (Saji et al. 2011).

#### Rifaximin

Rifaximin is a non-absorbed, gut-selective antibiotic with a low resistance profile that is commonly used to treat HE. It achieves high concentrations in the human intestine, where it is active against many enteropathogens (Goel et al. 2017; Hudson and Schuchmann 2019).

Rifaximin has always been a second-line drug in the treatment of HE, but there are no unified conclusions for treatment of MHE. Bajaj et al. demonstrated that over the 8-week period, MHE patients treated with rifaximin showed significant improvements in avoiding total driving errors, speeding, and illegal turns. Rifaximin also made improvements in the psychosocial dimension of the sickness impact profile and the anti-inflammatory cytokine IL-10 levels (Bajaj et al. 2011). However, Schulz et al. demonstrated that rifaximin therapy with and without lactulose over a period of 3 months does not affect bacterial composition (Schulz et al. 2019).

#### LOLA

LOLA may be administered orally or parenterally. The benefits of LOLA for the treatment of HE have been known for 50 years (Buyeverov et al. 2019; Kircheis and Luth 2019). However, in the treatment of MHE, there are no unified conclusions about the efficacy of LOLA. Buyeverov et al. demonstrated that fractional treatment with LOLA decreases level of ammonium ions in the blood and improves psychometric test results consequently. After 1 month of LOLA treatment, the CFF test was significantly improved and remained at that level for 9 months. After 5 months treatment, the NCT parameters reached their minimum values and remained at that level throughout the study. (Buyeverov et al. 2019). Alvares-da-Silva et al. showed that LOLA was useful in preventing further episodes of HE, but was no better in treating MHE (Alvares-da-Silva et al. 2014).

#### Lactulose

Lactulose is a disaccharide composed of galactose and fructose. As early as 1957, the prebiotic properties of lactulose were reported in both adults and infants. Due to promising outcomes, low price and high availability, Lactulose does not undergo cleavage by human gastrointestinal enzymes (Ruszkowski and Witkowski 2019; Schumann 2002). Lactulose may function as a prebiotic in the treatment of HE, so it can effectively modulate intestinal flora and reduce systemic level of ammonia. It significantly increases concentrations of bifidobacteria and lactobacilli; lactulose also can effectively inhibit urease-producing pathogenic bacteria like Enterobacteriaceae (Suraweera et al. 2016).

Psychometric tests improved in 75% of MHE patients after treatment with lactulose (El-Karaksy et al. 2017). In 2011, Luo et al. conducted a meta-analysis that demonstrated that lactulose significantly reduced the risk of no improvement in neuropsychological tests, the time required for the completion of the NCT-A, and the mean number of abnormal neuropsychological tests. Furthermore, lactulose prevented progression to overt HE, reduced blood ammonia levels, and improved health-related quality of life (Luo et al. 2011). Recent research has demonstrated the important role of lactulose in improving MHE. Singh et al. demonstrated that lactulose also leads to improvement in sleep disturbances in MHE patients (Singh et al. 2017).

#### Comparison

Probiotics, rifaximin, LOLA and lactulose can improve MHE, but they also have some weaknesses (Table 2). Probiotics have not undergone rigorous clinical trials, because they were considered more as health supplements or food. But more and more research demonstrated the safety and efficacy of probiotics for individual recipients were determined by host–microbe and host biochemical milieu interactions. So the potency of microbial species formulations, dosage, the immune response and strain-specific health effects must be studied (Hammerman et al. 2006; Sanders et al. 2010). Lactulose causes abdominal pain, diarrhea, intestinal malabsorption or flatulence. Lactulose may not be the optimal therapy for MHE patients due to poor compliance, cost, and adverse effects (Sharma et al. 2010). No significant adverse effects were observed with rifaximin and LOLA. However, the cost of rifaximin is high and patient compliance is not optimized (Butterworth and McPhail 2019; Chen et al. 2005; Goh et al. 2018; Lyon et al. 2017).

**Table 2:** Comparison of different kinds of drugs in improving MHE

	Advantage	Disadvantage
probiotic	☆ natural ☆well-tolerated therapy ☆excellent adherence	☆safety, effectiveness and immune response are unknown
rifaximin	☆antibiotic with low resistance ☆achieves high concentrations in intestine	☆cost is relative high ☆no significant side effect
LOLA	☆high availability ☆low price	☆no significant disadvantage
lactulose	☆high availability ☆low price ☆promising outcomes	☆flatulence ☆diarrhea ☆abdominal pain ☆intestinal malabsorption ☆poor compliance for the long-term treatment

In recent years, many scientists have compared the efficacy of these drugs in preventing and treating MHE, and there was no difference between probiotics and lactulose (Jiang et al. 2015). However, another study found that probiotics have long-term effects compared with lactulose (Shavakhi et al. 2014). Sidhu et al. conducted an RCT in which patients were randomized to treatment with lactulose or rifaximin. However, non-inferiority of rifaximin over lactulose could not be established and health-related quality of life was significantly improved in both groups (Sidhu et al. 2016). Zuo et al. demonstrated that treatment with rifaximin plus probiotics exhibited a different effect in MHE patients. The addition of probiotics to the treatment regimen distorted the distribution of bacteria and reduced *Streptococcus* abundance in the gut (Zuo et al. 2017). Butterworth et al. conducted a meta-analysis to compare the efficiency of LOLA, lactulose, probiotics and rifaximin in MHE patients. LOLA was comparable to the alternatives for both for slowing progression from MHE to HE and reversal of deficits in psychometric test scores (Butterworth and McPhail 2019).

Given the adverse effects of lactulose, cost of rifaximin and the safety of probiotics, LOLA appears to have beneficial effects in MHE, although its role in therapy is not clearly defined (Table 2).

## Discussion

MHE has serious consequences for quality of life, increasing the number of accidents, falls, hospitalizations and associated costs (Llansola et al. 2015). Ammonia or other liver abnormal metabolites can diffuse through the blood brain barrier in MHE patients. Then nerve cells, including astrocytes, microglial and neurons were damaged by DA, etc. Furthermore, cognitive areas, such as the hippocampus are affected (Fig. 1).

Timely and accurate discovery of cognitive impairment is the key to diagnosis of MHE. Psychometric tests, CFF, EEG and MRI are useful to evaluate cognitive function in an intuitive or abstract way. Psychometric tests are irreplaceable now. Compared with other psychometric tests, ANT is little influenced by age and education level. MRI, which can more accurately reflect changes in cognitive function, may be the best option in the future, if the problem of lack of detection accuracy of the measured signal can be resolved. CFF and EEG should be used alongside PHES (Table 1).

Timely correction of cognitive impairment is the key to treatment of MHE. One method is to prevent nerve cell injury, through improving the nutritional status of nerve cells, and blocking their injury by inflammatory mediators. Another method is to improve intestinal flora and reduce serum ammonia level. Among the drugs to improve intestinal flora, LOLA has few adverse effects and low cost, which may become the ideal choice in the future (Table 2).

## Abbreviations

ALFF: amplitude of low frequency fluctuation
ANT: animal naming test
CFF: critical flicker frequency
CHO: cholinesterase
DA: dopamine
EEG: electroencephalogram
eNCT: electronic number connection test
GABA: gama-aminobutyric acid
GLU: glutamine
HE: hepatic encephalopathy
ICT: inhibitory control test
IL: interleukin
LOLA: L-Ornithine L-aspartate
IR: insulin resistance
MD: mean diffusivity;
MHE: minimal hepatic encephalopathy
NCT: number connect test
MRI: magnetic resonance imaging
PG: prostaglandin
PHES: psychometric hepatic encephalopathy score
RCT: randomized controlled trial
TNF: tumor necrosis factor
